# Functional Characteristics of *Lactobacillus* and Yeast Single Starter Cultures in the Ripening Process of Dry Fermented Sausage

**DOI:** 10.3389/fmicb.2020.611260

**Published:** 2021-01-08

**Authors:** Yingli Liu, Zhen Wan, Kalekristos Woldemariam Yohannes, Qinglin Yu, Ziyan Yang, Hongyan Li, Jie Liu, Jing Wang

**Affiliations:** China-Canada Joint Lab of Food Nutrition and Health (Beijing), Beijing Advanced Innovation Center for Food Nutrition and Human Health (BTBU), Beijing Engineering and Technology Research Center of Food Additives, Beijing Technology & Business University (BTBU), Beijing, China

**Keywords:** starter culture, lipid oxidation, protein hydrolysis, volatile compound, functional differences, dry fermented sausage

## Abstract

Dry fermented sausage is popular among the world because of its rich nutrition and unique flavor. Starter cultures play an important role in the quality of dry fermented sausage. In this study, probiotics lactic acid bacteria *Lactobacillus delbrueckii* N102, *Latilactobacillus sakei* H1-5, *Debaryomyces hansenii* Y4-1, and *Wickerhamomyces anomalus* Y12-3 were isolated from food-borne materials. The physicochemical properties, microbial populations, TBARS, lipolysis, proteolysis, and volatile flavor compounds of dry fermented sausages with different starter cultures were evaluated comparatively during the ripening process. The results showed that both *L. delbrueckii* N102 and *L. sakei* H1-5 grow well and could rapidly reduce the pH value of the products. At the same time, they could significantly reduce the number of *Enterobacter putrefaciens*, so as to ensure the safety of the products. In addition, the strains N102 promoted the formation of flavor compounds 2,3-butanedione, 3-hydroxy-2-butanone, and carnosine, whereas taurine content of batch H1-5 was significantly increased, while yeast y4-1 and y12-3 could also grow faster in sausage and promoted the esters and alcohols formation such as ethyl acetate and linalool, with the formation of γ-aminobutyric acid by y4-1. Compared with lactic acid bacteria, yeasts showed to contribute more in flavor formation and effective inhibition of lipid oxidation. The starter cultures played different roles in flavor contribution and had obvious differentiation in the ripening process of dry fermented sausage.

## Introduction

Fermented sausage refers to the fermented meat products with stable microbial characteristics, typical fermentation flavor and long shelf life, which is made by mixing minced meat (often referring to pork or beef) with fat, sugar, salt, spices and other ingredients into the casing after microbial fermentation (Villani et al., 2007). Because of the variety and quantity of meat and raw materials, as well as the different fermentation and drying conditions, the sensory characteristics of products are diverse. Almost every country has its own traditional fermented sausage, For example: salami in Italy, dauerwurst in Germany, charqui in Spain, Chouriço de vinho in Portugal, and Harbin sausage in China. In order to improve the quality and safety of the final product and standardize the production process, exogenous microorganisms are often used as starter in the production of traditional or naturally dried fermented sausage. Studies have shown that the starter culture is mainly composed of *coagulase-negative cocci* (CNC), yeast, and *lactic acid bacteria* (LAB), such as *Latilactobacillus sakei*, *Latilactobacillus curvatus*, and *Lactiplantibacillus plantarum* (Cocolin et al., 2011).

Lactic acid bacteria plays a leading role in the sausage fermentation process. The main function of LAB is to reduce the pH of the matrix through production of lactic acid from the fermentation of sugars. A reduction in pH is necessary for fibrillar proteins to coagulate, resulting in improved firmness and cohesiveness of the final product, facilitating slicing (Drosinos et al., 2007). Montel et al. (1998) found that lactobacillus can give sausage special flavor by utilizing acetic acid, formic acid, and succinic acid generated by carbohydrate. Moreover, LAB species inhibit the multiplication of pathogenic and spoilage bacteria, mainly due to the production of organic acids or other antimicrobial metabolites, such as hydrogen peroxide, diacetyl, and peptides known as bacteriocins (Almeida da Costa et al., 2018). Bacteriocins have been shown to provide added control against pathogens in fermented sausages (Barbosa et al., 2015).

Yeast in dry fermented sausages produce a protection against the detrimental effect of oxygen and facilitate the drying process by protecting the sausage against fluctuation in humidity, which will produce changes in sausage appearance. Yeast can affect the color and flavor of sausage by their oxygen-scavenging and lipolytic activities, in addition to, it can utilize fermentation products such as lactic acid and contribute to increase more aroma compounds (Flores et al., 2015). Flores et al. (2004) first reported effect on VOCs and aroma by yeast starter cultures (*D. hansenii*) in fermented sausages due to the inhibition of lipid oxidation products (linear aldehydes) and promotion of ethyl ester compounds. Cano-Garcia et al. (2014a) found that inoculation of *D. hansenii* strains in fermented sausages can reduce lipid oxidation and produce flavor substances.

In the previous research, LAB and yeast with excellent characters were isolated from Chinese *Laminaria japonica* and traditional fermented foods (Chu et al., 2015; Liu et al., 2015). The purpose of these studies was to evaluate the improvement of the quality of dry fermented sausages and the contribution to flavor by inoculating them into sausages fermentation.

## Materials and Methods

### Bacterial Cultures and Culture Media

Autochthonous starter cultures are used for making dry fermented sausage. These strains included *L. delbrueckii* N102, *L. sakei* H1-5, *D. hansenii* Y4-1, and *W. anomalus* Y12-3 were previously isolated from Chinese *Laminaria japonica* and traditional fermented foods (Chu et al., 2015; Liu et al., 2015). *L. delbrueckii* N102 and *L. sakei* H1-5 have been proved to have strong acid production capacity and antimicrobial ability. *D. hansenii* Y4-1 and *W. anomalus* Y12-3 have been shown to contribute to good flavor formation. LAB were stored in glycerin and Man-Rogosa-Sharpe (MRS, Oxoid) broth medium mixture at −80°C until use, whereas yeasts were stored at −20°C on the yeast peptone dextrose (YPD, Oxoid) agar plate until use.

### Dry Fermented Sausage Manufacturing

The sausage was divided into following five batches: batch control without inoculation, batch N102 inoculated with about 10^7^ CFU g^–1^ of *L. delbrueckii* N102, batch H1-5 inoculated with about 10^7^ CFUg^–1^ of *L. sakei* H1-5, batch Y4-1 inoculated with about 10^6^CFUg^–1^ of *D. hansenii* Y4-1, batch Y12-3 inoculated with about 10^6^ CFUg^–1^ of *W. anomalus* Y12-3. The sausage formulation (200 g of meat-mixture for each sausage) include 70% lean pork, 30% fat, 3.5% NaCl, 0.2% glucose, 0.3% sucrose, 0.05% sodium ascorbate, 0.1% garlic powder, 0.3% white pepper, and 0.3% black pepper. Mix lean meat, fat, and ingredients with a blender at 4°C and add different starter cultures (Huang et al., 2020; Xiao et al., 2020).

Subsequently, the sausage mixture was filled into a natural casing (sheep small intestine) and placed in a fermentation chamber. All the sausages were fermented for 22 h at 22°C with 85% relative humidity (RH) and for 20 h at 20°C with 65% RH. Then the sausages are ripened for 18 h at 19°C with 67% RH, 22 h at 18°C with 69% RH, 18 h at 17°C with 71% RH, 21 h at 15°C with 73% RH, 21 h at 14°C with 76% RH, 16 h at 12°C with 77% RH and kept at 11°C with 37% RH until the end of 23 days.

From each batch, a 500 g portion of meat mixture (0 day) and three sausages at 5, 10, 16, and 23 days were randomly collected. 50 g portion of each sausage was minced and used for moisture and pH tests and a 25 g portion was taken for microbiological analysis. In addition, the remaining minced sausage was vacuum packaged and frozen at −20°C for subsequent analyses (TBARS, free fatty acids measurement, free amino acids measurement, sarcoplasmic proteins analysis, myofibrillar protein analysis). Also, 10 g sausage was wrapped in aluminum foil, vacuum packaged, and stored at −80°C for volatile compound analysis. Finally, sensory analysis was carried out at 23 days of the drying process. Results were expressed as the mean of three replicates per 100 g of dry matter at each processing time and batch.

### Measurement of pH Value

Measurement of pH was performed using a pH meter (Mettler Toledo Instruments Co., Ltd., Shanghai, China) in a homogenate prepared with 225 mL distilled water and 25 g samples.

### Determination of Water Content and Mobility

According to the method of Zhang et al. (2017), the change of water during sausage ripening was determined by LF-NMR.

### Measurement of Microbial Population

Shred dry fermented sausage samples (25 g) under aseptic conditions were added to 225 mL sterile saline and the samples were evenly oscillated. Microbiota were separated and counted using different selective media. The total number of bacteria was counted by incubation with Plate Count Agar (PCA, Oxoid) at 30°C for 72 h; LAB were counted in an anaerobic culture at 30°C for 48 h using Man-Rogosa-Sharpe agar (MRS, Oxoid)(Xiao et al., 2018); *Micrococci* and *Staphylococci* were counted on mannitol salt agar (MSA, Oxoid) at 30°C for 48–72 h; *Enterobacteriaceae* were counted on violet red bile glucose agar (VRBG, Oxoid) at 37°C for 24 h. The yeast was counted on yeast peptone dextrose agar (YPD, Oxoid) at 28°C for 5 days.

### Extraction of Myofibrillar and Sarcoplasmic Protein

The method of Mauriello et al. (2002), with some modifications to extract sarcoplasmic and myofibrillar proteins was used. The sample dilute with phosphate buffer (0.02 M, pH 6.5) was homogenized (4,000 rpm) for 4 min, then centrifuged at 10,000 × *g* for 20 min at 4°C. Supernatant filtered and sterilized was the sarcoplasmic protein extraction. The pretreatment of myofibrillar protein was the same as that of sarcoplasmic protein except that in the last step. The precipitate diluted with phosphate buffer (0.03 M, pH 6.5) containing 0.1% (v/v) Triton X-100 was homogenized (4,000 rpm) for 4 min, then centrifuged at 10,000 × *g* for 20 min at 4°C, for three times. The precipitate was suspended in phosphate buffer (0.1 M, pH 6.5, 0.7 M KI) with 0.02% (v/v) NaN_3_, homogenized (4,000 rpm) for 4 min at 4°C and centrifuged at 10,000 × *g* for 20 min at 4°C. The supernatant was sterilized by filtration, which was the myofibrillar protein extraction. All extracts are stored at 4°C until use.

Sarcoplasmic and myofibrillar proteins were analyzed by sodium dodecyl sulfate-polyacrylamide gel electrophoresis (SDS-PAGE) to evaluate the degree of proteolysis (Laemmli, 1970). SDS-PAGE was performed using a 12% acrylamide dissolution gel, a 6% acrylamide stacking gel and Mini-Protean Tetra System (Bio-Rad). The proteins used as standards were high molecular weight standard protein Marker (Sigma, MW-SDS-200). The polygels were scanned with a gel imager (Bio-Rad).

### Measurement of Free Amino Acids

Free amino acids were extracted by the method of Casaburi et al. (2007) and determined reverse phase by HPLC as described and determined by Bidlingmeyer et al. (1987).

### Thiobarbituric Acid Reactive Substances

The TBARS of sausages was analyzed according to the method of Wang and Xiong (2005). The TBARS value was expressed as mg of malondialdehyde/100 g sausage.

### Measurement of Free Fatty Acids

The extraction of lipids and the separation of free fatty acids were carried out by referring to the method of Bligh and Dyer (1959). After methyl esterification of free fatty acids, the determination of free fatty acid content and composition by gas chromatography was carried out according to the method of Navarro et al. (1997).

### Analysis of Volatile Compounds

Volatile compounds were extracted by solid-phase micro-extraction (SPME) (Wen et al., 2019). Four grams of sausage was placed in a 20 ml headspace extraction flask, equilibrated at 60°C for 30 min and then extracted with a 50 μm layer DVD/PDMS for 30 min at 60°C. After sample extraction was complete, the fibers were drawn into the needle and transferred to the injection of the GC-MS system for desorption at 250°C for 5 min. GC-MS analysis was performed on model 7890A of Agilent Technologies (Paolo Alto, CA, United States) gas chromatograph, and model 5975C of mass spectrometry detector was selected. GC conditions: TRACE TR-5 GC column (30 m, 0.25 mm × 0.25 μm); the initial column temperature was maintained at 40°C for 5 min, then raised to 100°C at 5°C/min and held isothermal 10 min, then from 100°C to 180°C at a rate of 5°C/min, and finally, the temperature was raised to 240°C for 10 min a rate of 15°C/min. The carrier gas is helium and the flow rate was 1 mL min^–1^. MS conditions: ionization voltage 70 eV; ion source temperature 280°C; mass scan range: 30–300 mass units (Martin et al., 2003). The peak components were determined through the search of NIST14 standard library, and the flavor component was determined by the matching degree greater than 80%. The relative content was expressed as the percentage of the peak area of each component to the total peak.

### Sensory Analysis

Twelve experienced panelists were chosen to evaluate the sensory properties. The evaluations were conducted in a sensory panel room at 25°C. The sausage sample was cut into slices (3 mm thick) and placed in a white plastic dish. The samples were blind-coded with 3-digit random numbers and evaluated three times. Unsalted crackers and water were used to cleanse the palate between samples. The following sensory characteristics were evaluated: color, aroma, chewiness, acid taste, and overall acceptability. The intensity or degree of an attribute was expressed by a seven-point descriptive scale from 1 (low intensity) to 7 (high intensity): color (7 = red and shiny; 1 = dark and dull), aroma (7 = strong; 1 = light), chewiness (7 = hard; 1 = soft), acid taste (7 = strong acid taste; 1 = light acid taste), and overall acceptability (7 = high; 1 = low).

### Statistical Analysis

All experimental data were repeated three times and the results were expressed as mean and standard deviation. The data were analyzed by one-way ANOVA using the SPSS 13.0 software for Windows (SPSS Chicago, IL, United States) and means were compared by Duncan’s multiple comparison range test. Values were considered significantly different at *P* < 0.05. Biplot based on principal component analysis (PCA) was performed using the SIMCA software (version 14.1, Umeå, Sweden) for volatile compounds data.

## Results

### Effects of Different Starter Cultures on pH Value Changes of Dry Fermented Sausage

**FIGURE 1:**
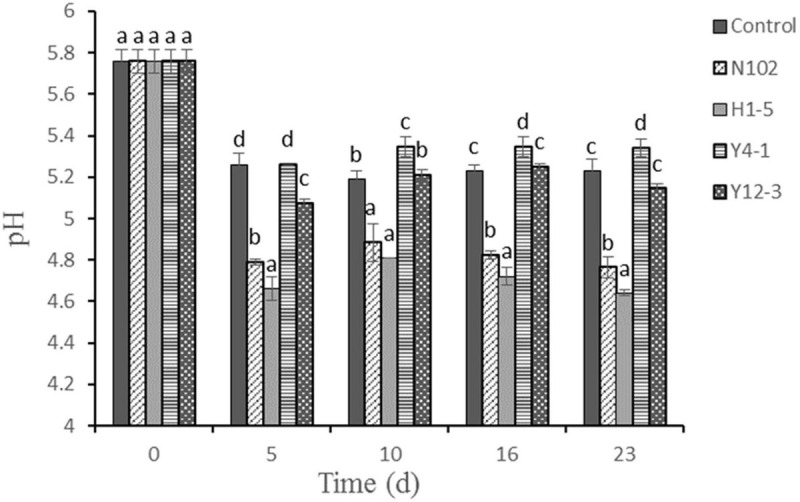
Evolution of pH value during the ripening of the different samples of sausage non-inoculated and inoculated with various starter cultures. Error bars refer to the standard deviations obtained from triplicate sample analysis. Different letters (a–d) indicate significant differences among different batches at the same fermentation time (*P* < 0.05). N102, *L. delbrueckii*; H1-5, *L. sakei*; Y4-1, *D. hansenii*; Y12-3, *W. anomalus*.

As shown in Figure 1, the pH values of fermented sausages significantly decreased at the first 5 days in all batches (*P* < 0.05). The initial pH value was about 5.8. After 12 days fermentation, the pH of control, N102, H1-5, Y4-1, and Y12-3 were reduced to 5.23, 4.76, 4.64, 5.30, and 5.14, respectively. The pH values of N102 and H1-5 batches were lower than the control throughout the sausage ripening process (*P* < 0.05) and its pH was below 5.0. However, the pH of the batch inoculated with yeast was not significant compared with the control during sausage ripening. The pH of all batches increased on the 10th to 16th day of drying, and the change in pH decreased slightly after 23 days fermentation.

### Effects of Different Starter Cultures on Water State of Dry Fermented Sausage

**FIGURE 2:**
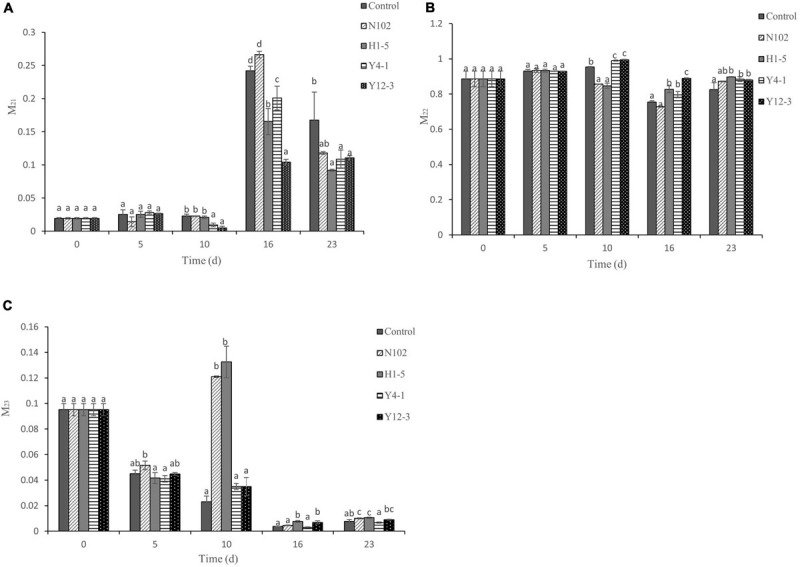
Relative area percentages of hydration water **(A)**, immobilized water **(B)**, and free water **(C)** populations. Error bars refer to the standard deviations obtained from triplicate sample analysis. Different letters (a–d) indicate significant differences among different batches at the same fermentation time (*P* < 0.05). N102, *L. delbrueckii*; H1-5, *L. sakei*; Y4-1, *D. hansenii*; Y12-3, *W. anomalus.*

The state of water in sausages was investigated by low-field nuclear magnetic resonance (LF-NMR). Figure 2 shows the change in the percentage of water state at different fermentation time during the dry fermented sausage making, the proportion of immobilized water is up to 88.55%, the content of free water is 9.52%, and the content of bound water is at least 1.93%. The proportion of bound water in each batches increased with the drying of the sausage. In the first 10 days, the proportion of bound water in each batches did not exceed 2.28%, while in the late drying stage, the proportion accounted for more than 20% in all samples, of which, in 16 days and 23 days of ripening, the content of bound water of the batch inoculated with H1-5, Y4-1, and Y12-3 was significantly lower than that of the control (*P* < 0.05). In the first 10 days, the proportion of immobilized water was still the highest. In the late stage of drying, the proportion of immobilized water of the batches inoculated with H1-5, Y4-1, and Y12-3 was significantly higher than that in the control (*P* < 0.05). The proportion of free water in each batch decreased from 9.52% at the beginning to less than 1% at the end.

### Effects of Different Starter Cultures on Microbial Population of Dry Fermented Sausage

**FIGURE 3:**
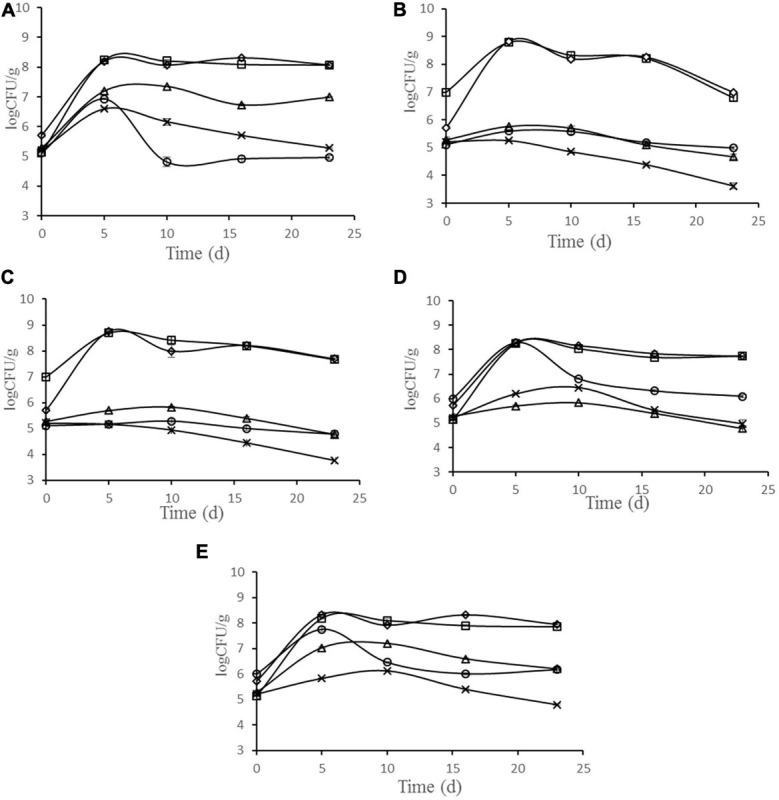
Evolution of microbial populations during the ripening of different samples of sausages non-inoculated and inoculated with various starter cultures. Error bars refer to the standard deviations obtained from triplicate sample analysis. **(A)** control (uninoculated) sausage; **(B)** sausage inoculated with the strain *L. delbrueckii* N102; **(C)** sausage inoculated with the strain *L. sakei* H1-5; **(D)** sausage inoculated with the strain *D. hansenii* Y4-1; **(E)** sausage inoculated with the strain *W. anomalus* Y12-3. (□) LAB; (△) *Micrococci* and *Staphylococci*; (×) *Enterobacteriaceae*;(○)Yeast; (♢)Total bacterial plate.

The changes in microbiota are shown in Figure 3. During the ripening of sausage, LAB were dominant bacteria in all batches, and the number was much higher than other bacteria. The number of LAB reached the maximum at 8.79, 8.69, 8.25, 8.18, and 8.23 log cfu g^–1^ after 5 days fermentation, in the batches inoculated with starter cultures N102, H1-5, Y4-1, Y12-3, and the control, respectively. The growth rate of *Micrococci* and *Staphylococci* increased in the early stage of processing and showed a downward trend in the later stage. In batches N102 and H1-5, the numbers of *Micrococci* and *Staphylococci* were lower than other batches, probably because LAB lowered the pH of the sausage and inhibited their growth. As spoilage bacteria in meat products, the number of *Enterobacteriaceae* showed a downward trend in all batches. In batches N102 and H1-5, the number of *Enterobacteriaceae* was significantly lower than that of other batches, indicating that *L. delbrueckii* N102 and *L. sakei* H1-5 had a significant inhibitory effect on the growth of *Enterobacteriaceae*. The initial yeast quantity for batches Y4-1 and Y12-3 was 6 log cfu/g, which was consistent with the quantity of starter added. The number of yeasts in batches Y4-1 and Y12-3 showed an upward trend in the early stage of processing, and reached the maximum values of 8.26 and 7.75 log cfu/g on the 5th day, which was significantly higher than other batches of about 5 log cfu/g. At end of sausage ripening, the amount of yeast then showed a downward trend, with the number of final products being about 6 log cfu g^–1^ of the batch inoculated with Y4-1 and Y12-3, while the number of other batches was about 4.95 log cfu g^–1^.

### Effects of Different Starter Cultures on Proteolysis of Dry Fermented Sausage

**FIGURE 4:**
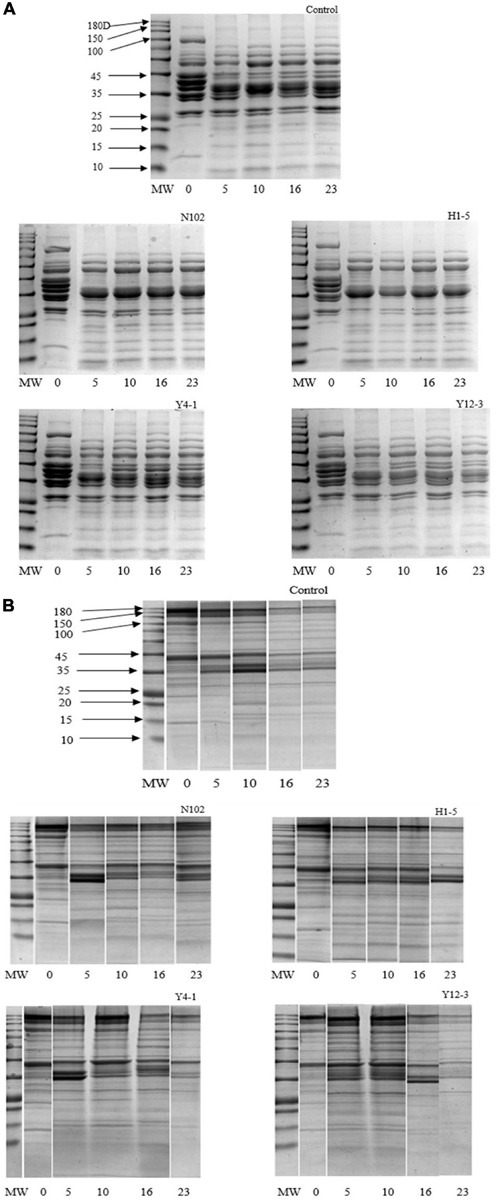
SDS-PAGE analyses of sarcoplasmic proteins **(A)** and myofibrillar protein **(B)** degradation during the ripening of the different samples of sausages non-inoculated and inoculated with various starter cultures. MW refers to the molecular weights of a protein standard; 0–23 refer to fermentation time (days). N102, *L. delbrueckii*; H1-5, *L. sakei*; Y4-1, *D. hansenii*; Y12-3, *W. anomalus*.

Degradation of myofibrillar and sarcoplasmic proteins in each batch was analyzed by SDS-PAGE technique. The degradation of sarcoplasmic proteins is shown in the Figure 4A. In the batch inoculated with N102 and H1-5, from the fermentation stages to the ripening of sausage, the protein bands of 157, 97, 45, and 29 kD gradually weakened and disappeared during the fermentation and ripening stage. Compared to the other three batches, the 25 kD protein band of the N102 and H1-5 batches were weaker in color. The protein bands of only 97 and 45 kD were weakened in the control and the Y4-1 and Y12-3 batches. The low protein bands below 20 kD of the batch inoculated Y12-3 were weaker in color than the other batches. As shown in Figure 4B, actin and myosin were significantly degraded in each batch, as well as the heavy chain of myosin.

### Effects of Different Starter Cultures on Free Amino Acids of Dry Fermented Sausage

**FIGURE 5:**
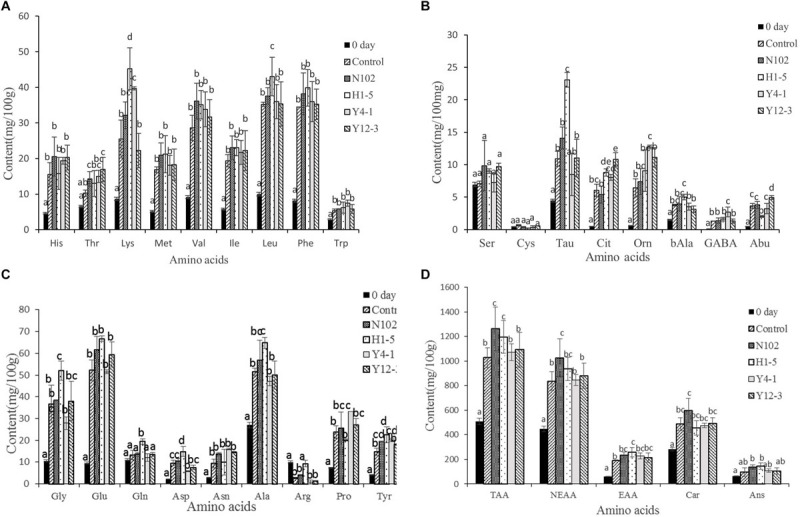
Free amino acids content of the different samples of sausages non-inoculated and inoculated with various starter cultures. Concentration of His, Thr, Lys, Met, Val, Ile, Leu, Phe and Trp **(A)**; Concentration of Ser, Cys, Tau, Cit, Orn, bAla, GABA and Abu **(B)**; the concentration of Gly, Glu, Gln, Asp, Asn, Ala, Arg, Pro and Tyr **(C)**; Concentration of TAA, NEAA, EAA, Car and Ans **(D)**. Error bars refer to the standard deviations obtained from triplicate sample analysis, expressed as mg free amino acid/100 g dry-matter sample. Different letters (a–d) indicate significant differences among the values (*P* < 0.05). TAA, total amino acids; NEAA, nonessential amino acids; EAA, essential amino acid. N102, *L. delbrueckii*; H1-5, *L. sakei*; Y4-1, *D. hansenii*; Y12-3, *W. anomalus*.

In order to evaluate the effect of the starter cultures on proteolysis, the free amino acids in the meat mixture of 0 day and in all batches of sausages ripening were determined. The results are shown in Figure 5. The main amino acids in the meat mixture used for sausage processing were carnosine, anserine, leucine, alanine, glycine; and the content of cysteine, ornithine, and citrulline was at least about 0.5 mg/100 g meat. At the end of ripening, the total amino acid content of each batch was significantly higher than the sausage at the beginning of processing (*P* < 0.05) (from 509.38 at 0 days to 1028.52, 1263.04, 1196.57, 1072.65, 1093.63 mg/100 g sausage in control sausage and in the batches inoculated with N102, H1-5, Y4-1, and Y12-3, respectively, at end of ripening). At the same stage, the total amino acid content of the batch inoculated with N102, H1-5, Y4-1, and Y12-3 was higher than that of the control(*P* < 0.05), indicating that these starter cultures may contribute to the formation of free amino acids. At end of sausage ripening, the content of threonine, tyrosine and carnosine in the batch inoculated with N102 was significantly higher than that in the control (*P* < 0.05); the contents of taurine, citrulline, glycine, glutamine, aspartic acid, alanine, tyrosine, lysine, and leucine in the batch inoculated with H1-5 was significantly higher than that in the control (*P* < 0.05); In addition, the contents of tyrosine, taurine and leucine in batch H1-5 were significantly higher than those in other batches (*P* < 0.05). Lysine, proline, tyrosine, citrulline, ornithine, and γ-aminobutyric acid in the batch inoculated with Y4-1 were significantly higher than that in the control (*P* < 0.05), Moreover, the contents of ornithine and proline were significantly higher than those of other batches (*P* < 0.05). The content of 2-aminobutyric acid, citrulline, and threonine in the batch inoculated with Y12-3 was significantly higher than that in the control (*P* < 0.05). However, the content of arginine in each batch at the end of fermentation was lower than that at 0 days. In general, Val, Leu, Phe, Glu, Ala, Car, and Ans were the most abundant amino acids in the all batches.

### Effects of Different Starter Cultures on Free Fatty Acids of Dry Fermented Sausage

The effects of various starter cultures on lipid hydrolysis were evaluated by determining the content of free fatty acids. The results are shown in Table 1. The content of saturated fatty acids (C14:0, C16:0, and C18:0), monounsaturated fatty acids (C16:1, C18:1), polyunsaturated fatty acids (C18:2, C18:3, C20:2, C20:3, and C20:4) were determined at 0 days and the ripening period of all batches. At the end of ripening, the total free fatty acids content was significantly higher than the free fatty acids content of the sausage at the beginning of processing (*P* < 0.05)(from 257.00 at 0 days to 1037.97, 685.64, 1019.21, 873.21, and 736.73 mg/100 g sausage in control sausage and in the batches inoculated with N102, H1-5, Y4-1, and Y12-3, respectively, at end of ripening). Palmitic acid, oleic acid, and linoleic acid were the three highest free fatty acids in all samples. All samples had the highest monounsaturated fatty acid content, followed by saturated fatty acids and polyunsaturated fatty acids at the end of ripening.

**TABLE 1:** Changes in free fatty acids (FFA) of the different samples of sausages non-inoculated and inoculated with various starter cultures.

**Time (day)**	**0**	**28**				
**FFA**	**Control**	**Control**	**N102**	**H1-5**	**Y4-1**	**Y12-3**
Myristic	3.79 ± 1.01^*a*^	13.63 ± 1.27^*d*^	8.33 ± 0.64^*b*^	11.71 ± 0.60^*c*^	11.54 ± 0.08^*c*^	9.38 ± 1.47^*bc*^
Palmitic	73.28 ± 25.91^*a*^	202.56 ± 10.53^*d*^	143.48 ± 0.93^*b*^	194.92 ± 6.99^*cd*^	179.63 ± 12.54^*bcd*^	150.37 ± 35.20^*bc*^
Stearic	40.42 ± 19.59^*a*^	87.25 ± 3.11^*b*^	67.31 ± 0.92^*ab*^	87.55 ± 4.09^*b*^	80.80 ± 7.64^*b*^	66.04 ± 14.83^*ab*^
Arachidic	2.08 ± 0.81^*a*^	10.19 ± 0.63^*c*^	6.81 ± 1.25^*b*^	10.75 ± 0.42^*c*^	8.82 ± 0.21^*bc*^	6.99 ± 1.79^*b*^
Palmitoleic	4.39 ± 0.83^*a*^	23.41 ± 1.39^*c*^	14.14 ± 1.48^*b*^	23.38 ± 0.04^*c*^	19.50 ± 0.69^*c*^	18.35 ± 4.56^*bc*^
Octadecenoic	90.72 ± 26.09^*a*^	445.10 ± 20.24^*c*^	278.99 ± 25.22^*b*^	439.35 ± 8.57^*c*^	357.55 ± 19.46^*bc*^	304.23 ± 6.62^*b*^
Linoleic	38.62 ± 7.25^*a*^	227.69 ± 15.05^*c*^	148.29 ± 11.12^*b*^	225.96 ± 0.86^*c*^	190.02 ± 5.09^*bc*^	159.31 ± 6.74^*b*^
g-linolenic	0.50 ± 0.07^*a*^	4.01 ± 0.57^*d*^	2.51 ± 0.53^*b*^	3.58 ± 0.18^*cd*^	3.43 ± 0.20^*bcd*^	2.97 ± 0.35^*bc*^
Eicosadienoic	1.67 ± 0.49^*a*^	9.32 ± 0.83^*c*^	7.13 ± 1.74^*bc*^	9.40 ± 0.42^*c*^	7.38 ± 0.14^*bc*^	5.82 ± 1.27^*b*^
Eicosatrienoic	0.00^*a*^	0.75 ± 0.01^*d*^	0.46 ± 0.00^*b*^	0.57 ± 0.03^*c*^	0.56 ± 0.02^*c*^	0.00^*a*^
Arachidonic	2.64 ± 0.13^*a*^	14.08 ± 1.22^*c*^	8.19 ± 0.29^*b*^	12.02 ± 0.84^*bc*^	13.97 ± 0.10^*c*^	13.27 ± 4.95^*bc*^
SFA	119.56 ± 47.32^*a*^	313.63 ± 15.53^*c*^	225.93 ± 3.73^*b*^	304.94 ± 12.09^*bc*^	280.80 ± 20.30^*bc*^	232.79 ± 53.29^*bc*^
MUFA	95.11 ± 26.92^*a*^	468.50 ± 21.63^*c*^	293.13 ± 26.70^*b*^	462.73 ± 8.60^*c*^	377.05 ± 20.15^*bc*^	322.58 ± 11.18^*b*^
PUFA	42.33 ± 6.27^*a*^	255.84 ± 17.66^*c*^	166.58 ± 13.68^*b*^	251.54 ± 1.13^*c*^	215.36 ± 5.16^*bc*^	181.36 ± 13.31^*b*^
Total FFA	257.00 ± 80.51^*a*^	1037.97 ± 54.82^*c*^	685.64 ± 44.11^*b*^	1019.21 ± 21.82^*c*^	873.21 ± 45.61^*bc*^	736.73 ± 77.78^*b*^

*The results are expressed as mg/100 g dry matter and are means of three replicates ± standard deviations. Different lowercase letters (a–d) indicate significant differences among the values (*P* < 0.05). SFA, saturated fatty acids; MUFA, monounsaturated fatty acids; PUFA, polyunsaturated fatty acids. N102, *L. delbrueckii*; H1-5, *L. sakei*; Y4-1, *D. hansenii*; Y12-3, *W. anomalus*.*

### Effects of Different Starter Cultures on Lipid Oxidation of Dry Fermented Sausage

**FIGURE 6:**
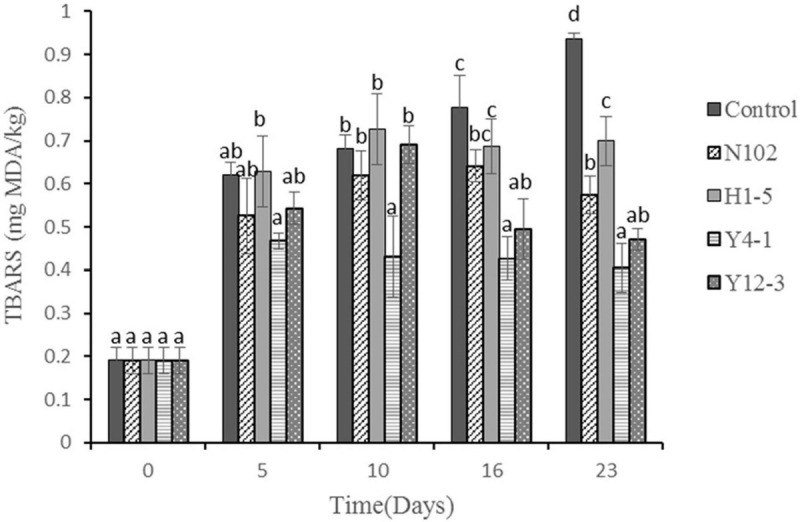
Thiobarbituric acid reactive substances (TBARS) (mg MDA/kg meat) during the ripening of the different samples of sausages non-inoculated and inoculated with various starter cultures. Different letters (a–d) indicate significant differences among different batches at the same fermentation time (*P* < 0.05). N102, *L. delbrueckii*; H1-5, *L. sakei*; Y4-1, *D. hansenii*; Y12-3, *W. anomalus*.

TBARS evaluates the degree of oxidation of sausages during processing by measuring the amount of secondary products (such as malondialdehyde) produced during the oxidation process. The results are shown in Figure 6. In all batches, the TBARS value gradually increased with the sausage ripening time, reaching a maximum of 0.94, 0.57, 0.70, 0.41, and 0.47 mg MDA/kg sausage in control sausage, N102, H1-5, Y4-1, and Y12-3, respectively, at end of ripening. The TBARS value of the batch added to the starter cultures was significantly lower than that of the control (*P* < 0.05), indicating that the starter cultures could inhibit the oxidation of lipids. Among them, the TBARS value of the batch inoculated with Y4-1 and Y12-3 was lower than that of the other batch, indicating that the yeast may have an inhibitory effect on lipid oxidation.

### Effects of Different Starter Cultures on Flavor Compounds of Dry Fermented Sausage

A total of 59 flavor substances were detected in 5 batch samples, including 29 substances in the control, 25 substances in the batch inoculated with N102, 24 flavor substances in the batch inoculated with H1-5, and 40 substances in the batches inoculated with Y4-1 and Y12-3, respectively. According to the results shown in Table 2, the main volatile substances can be divided into ketones, acids, esters, aldehydes, alcohols, terpenes and some other substances. Terpenes are mainly produced by adding black pepper, white pepper and garlic powder to sausage (Guadayol et al., 1997). The 2, 3-butanediol was detected in all five batches of sausages with high content. Batch N102 and batch H1-5 detected less alcohol content and categories than the control and yeast batches. The ethanol content of batch Y4-1 was significantly higher than that of other batches. 2-phenylethanol was detected in batch Y4-1 and batch Y12-3, and linalool was detected in batch Y12-3, which had an important effect on the flavor of sausage. Butyric acid was detected in all batches and the batches inoculated with Y4-1 and Y12-3 had higher levels of butyric acid, which is an important flavor substance. The isobutyric acid was detected in the batch inoculated with Y4-1 and 2-methylbutyric acid was detected only in the batch inoculated with N102. Only ethyl lactate and 2-methyl-1-butylethyl ester were detected in the batch inoculated with H1-5and N102, and the total ester substances in this two batches were lower than those in the control. The ethyl acetate content of the batches inoculated Y4-1 and Y12-3 was significantly higher than that of the control. Further, seven types of ester substances were detected in the Y4-1 batch, which indicated the strain Y4-1 contributed to the formation of ester substances. The content of ethyl lactate and ethyl isovalerate in the batch Y12-3 was higher than that in the control. The presence of benzaldehyde was detected in all samples, which is a unique flavor component of the fermented meat product. In addition to the batch inoculated with Y4-1, the other batches had the highest content of hexanal, which had an apple aroma and improved the flavor. The content of hexanal in the batch H1-5 and batch Y12-3 was higher than that in the control. Only 3-hydroxy-2-propanone was detected in the control, which did not appear in other batches. The contents of 2,3-butanedione and 3-hydroxy-2-butanone in the batches inoculated with N102 and H1-5 were very high. The presence of 3-hydroxy-2-butanone was detected in the batches inoculated with Y4-1 and Y12-3.

**TABLE 2:** Volatile compounds identified and quantified by SPME–GC–MS of the different samples of sausages non-inoculated and inoculated with various starter cultures.

**Volatile compounds**	**Strains**				
	**Control**	**N102**	**H1-5**	**Y4-1**	**Y12-3**
**Aldehydes**					
Benzaldehyde	0.44 ± 0.02^*a*^	0.45 ± 0.02^*a*^	0.34 ± 0.02^*b*^	0.17 ± 0.01^*c*^	0.46 ± 0.02^*a*^
Hexanal	1.62 ± 0.08^*c*^	0.78 ± 0.04^*d*^	2.56 ± 0.13^*b*^	n. d.	4.43 ± 0.22^*a*^
Nonana	0.10 ± 0.01^*a*^	n. d.	n. d.	n. d.	0.08 ± 0.01^*a*^
Heptanal	n. d.	0.12 ± 0.01^*b*^	n. d.	n. d.	0.21 ± 0.01^*a*^
Total	2.16 ± 0.11^*c*^	1.35 ± 0.07^*d*^	2.9 ± 0.15^*b*^	0.17 ± 0.01^*e*^	5.18 ± 0.26^*a*^
**Alcohols**					
1-pentanol	0.19 ± 0.01^*b*^	0.24 ± 0.01^*b*^	0.15 ± 0.01^*b*^	n. d.	0.31 ± 0.02^*a*^
4-isopropyltoluene	2.65 ± 0.13^*a*^	n. d.	n. d.	1.51 ± 0.08^*c*^	1.78 ± 0.10^*b*^
1-hexanol	0.11 ± 0.01^*a*^	n. d.	n. d.	0.03	0.13 ± 0.01^*a*^
2-ethyl-1-hexanol	0.14 ± 0.01^*a*^	0.06 ± 0.01^*c*^	n. d.	0.03^*d*^	0.09^*b*^
2,3-butanediol	5.31 ± 0.27^*a*^	1.64 ± 0.08^*c*^	1.76 ± 0.09^*c*^	5.77 ± 0.2^*a*^	3.6 ± 0.18^*b*^
Isobutanol	1.19 ± 0.06^*a*^	n. d.	n. d.	0.08^*c*^	0.74 ± 0.04^*b*^
3-methyl-1-butanol	n. d.	0.58 ± 0.03^*c*^	0.38 ± 0.02^*d*^	0.89 ± 0.04^*a*^	0.81 ± 0.04^*b*^
Ethanol	n. d.	n. d.	0.46 ± 0.02^*c*^	14.28 ± 0.71^*a*^	1.55 ± 0.08^*b*^
2-phenylethanol	n. d.	n. d.	n. d.	0.04 ± 0.01^*a*^	0.05 ± 0.01^*a*^
Linalool	n. d.	n. d.	n. d.	n. d.	0.12 ± 0.01^*a*^
Total	9.59 ± 0.48^*b*^	2.52 ± 0.13^*c*^	2.75 ± 0.14^*c*^	22.63 ± 1.13^*a*^	9.18 ± 0.46^*b*^
**Ketones**					
3-hydroxy-2-butanone	9.15 ± 0.46^*a*^	n. d.	n. d.	n. d.	n. d.
2-butanone	n. d.	1.30 ± 0.07^*a*^	n. d.	n. d.	n. d.
2,3-butanedione	n. d.	21.50 ± 1.08^*a*^	9.36 ± 0.47^*a*^	n. d.	n. d.
2,3-octanedione	n. d.	37.26 ± 1.86^*a*^	27.06 ± 1.35^*b*^	3.83 ± 0.19 ^d^	4.77 ± 0.24^*c*^
2-heptenone	n. d.	n. d.	n. d.	n. d.	0.03
Total	9.15 ± 0.46 ^c^	60.06 ± 3.00 ^a^	36.42 ± 1.82 ^b^	3.83 ± 0.19 ^e^	4.8 ± 0.24 ^d^
**Acids**					
Acetic acid	21.1 ± 1.06 ^a^	16.71 ± 0.84 ^b^	23.21 ± 1.16 ^a^	21.73 ± 1.09 ^a^	22.29 ± 1.11 ^a^
Propionic acid	0.3 ± 0.02 ^a^	n. d.	n. d.	0.18 ± 0.01 ^c^	0.24 ± 0.01 ^b^
Butyric acid	0.52 ± 0.03 ^c^	0.34 ± 0.02 ^d^	0.53 ± 0.03 ^c^	0.62 ± 0.03 ^b^	0.83 ± 0.04 ^a^
Hexanoic acid	0.25 ± 0.01 ^c^	0.19 ± 0.01 ^d^	0.13 ± 0.01 ^e^	2.41 ± 0.12 ^a^	0.36 ± 0.02 ^b^
Isobutyric acid	n. d.	0.41 ± 0.02 ^b^	0.41 ± 0.02 ^b^	2.17 ± 0.11 ^a^	n. d.
2-methylbutanoic acid	n. d.	0.98 ± 0.05 ^a^	n. d.	n. d.	n. d.
Isovaleric acid	n. d.	n. d.	0.94 ± 0.05 ^a^	n. d.	n. d.
Pentanoic acid	n. d.	n. d.	n. d.	0.02	n. d.
Octanoic acid	n. d.	n. d.	n. d.	n. d.	0.08
Total	22.17 ± 1.11 ^c^	18.63 ± 0.93 ^d^	25.22 ± 1.26^*b*^	27.13 ± 1.36 ^a^	23.8 ± 1.19 ^c^
**Esters**					
Ethyl acetate	13.66 ± 0.68 ^b^	n. d.	n. d.	21.64 ± 1.08 ^a^	22.01 ± 1.10 ^a^
Ethyl 3-methylbutanoate	0.27 ± 0.01 ^b^	n. d.	n. d.	0.28 ± 0.01 ^b^	0.70 ± 0.04 ^a^
Ethyl hexanoate	0.12 ± 0.01 ^c^	n. d.	n. d.	0.02 ^b^	0.20 ± 0.01 ^a^
Ethyl lactate	0.20 ± 0.01 ^d^	0.20 ± 0.01 ^d^	0.37 ± 0.02 ^b^	0.28 ± 0.01 ^c^	0.93 ± 0.05 ^a^
2-methyl-1-butyl acetate	n. d.	n. d.	4.82 ± 0.24 ^a^	0.73 ± 0.04 ^b^	n. d.
Ethyl pentanoate	n. d.	n. d.	n. d.	0.73 ± 0.04 ^a^	n. d.
Methylbutyl acetate	n. d.	n. d.	n. d.	0.26 ± 0.01 ^a^	n. d.
Total	14.25 ± 0.71 ^b^	0.2 ± 0.01 ^d^	5.19 ± 0.26 ^c^	23.94 ± 1.19 ^a^	23.84 ± 1.19 ^a^
**Terpenes**					
α-pinene	0.83 ± 0.04 ^c^	1.32 ± 0.07 ^b^	1.44 ± 0.07 ^a^	0.58 ± 0.03 ^d^	n. d.
β-pinene	1.40 ± 0.07 ^a^	1.03 ± 0.05 ^b^	1.45 ± 0.07 ^a^	0.88 ± 0.04 ^c^	0.92 ± 0.05 ^c^
δ-3-carene	17.91 ± 0.90 ^a^	7.53 ± 0.38 ^d^	11.30 ± 0.57^*b*^	10.01 ± 0.50 ^c^	11.36 ± 0.57^*b*^
Sabinene	1.79 ± 0.09 ^a^	0.27 ± 0.01 ^d^	0.59 ± 0.03 ^c^	0.77 ± 0.04 ^b^	0.74 ± 0.04 ^b^
D-limonene	8.18 ± 0.41 ^a^	3.07 ± 0.1^*e*^	3.58 ± 0.18 ^d^	3.96 ± 0.20 ^c^	5.47 ± 0.27 ^b^
Terpinolene	0.18 ± 0.01 ^a^	n. d.	n. d.	0.04	n. d.
α-terpinene	n. d.	n. d.	n. d.	0.03 ^a^	n. d.
α-cubebene	0.14 ± 0.01 ^a^	0.04	n. d.	0.03 ^b^	0.12 ± 0.01 ^a^
β-caryophyllene	4.74 ± 0.24 ^a^	1.59 ± 0.08 ^d^	1.25 ± 0.06 ^e^	2.28 ± 0.11 ^c^	3.92 ± 0.10 ^b^
β-selinene	0.13 ± 0.01 ^a^	n. d.	n. d.	n. d.	0.15 ± 0.01 ^a^
Alloaromadendrene	n. d.	n. d.	n. d.	0.04 ^b^	0.07 ^a^
Isocaryophyllene	n. d.	n. d.	n. d.	n. d.	0.14 ± 0.01 ^a^
1-isopropenyl-4-methylbenzene	n. d.	n. d.	n. d.	n. d.	0.08 ^a^
δ-elemene	n. d.	n. d.	n. d.	n. d.	0.35 ± 0.02 ^a^
Caryophyllene	n. d.	n. d.	0.70 ± 0.04 ^a^	n. d.	n. d.
Phenylethylene	n. d.	n. d.	n. d.	0.12 ± 0.02 ^a^	n. d.
Total	35.3 ± 1.77 ^a^	14.85 ± 0.74 ^e^	20.31 ± 1.02^*c*^	18.74 ± 0.94^*d*^	23.32 ± 1.17^*b*^
**Others**					
Toluene	2.03 ± 0.10^*b*^	1.4 ± 0.07 ^c^	0.69 ± 0.03 ^e^	1.07 ± 0.05 ^d^	2.43 ± 0.12 ^a^
2-acetyl-1-pyrroline	0.33 ± 0.02 ^a^	n. d.	n. d.	0.02 ^c^	0.03 ^b^
4-isopropyltoluene	n. d.	0.92 ± 0.05 ^b^	1.09 ± 0.05 ^a^	0.77 ± 0.04 ^c^	n. d.
Dimethyl trisulfide	n. d.	0.08 ^a^	n. d.	0.05 ^b^	0.08 ^a^
4-methylphenol	n. d.	n. d.	n. d.	n. d.	0.02 ^a^
2,6-dimethyl pyrazine	n. d.	n. d.	n. d.	0.03 ^a^	n. d.
Total	2.36 ± 0.12 ^b^	2.4 ± 0.12 ^b^	1.78 ± 0.08 ^d^	1.94 ± 0.09 ^c^	2.56 ± 0.12 ^a^

*The results are means of three replicates ± standard deviations. n. d., not detected.*

### Principal Component Analysis of Volatile Compounds

**FIGURE 7:**
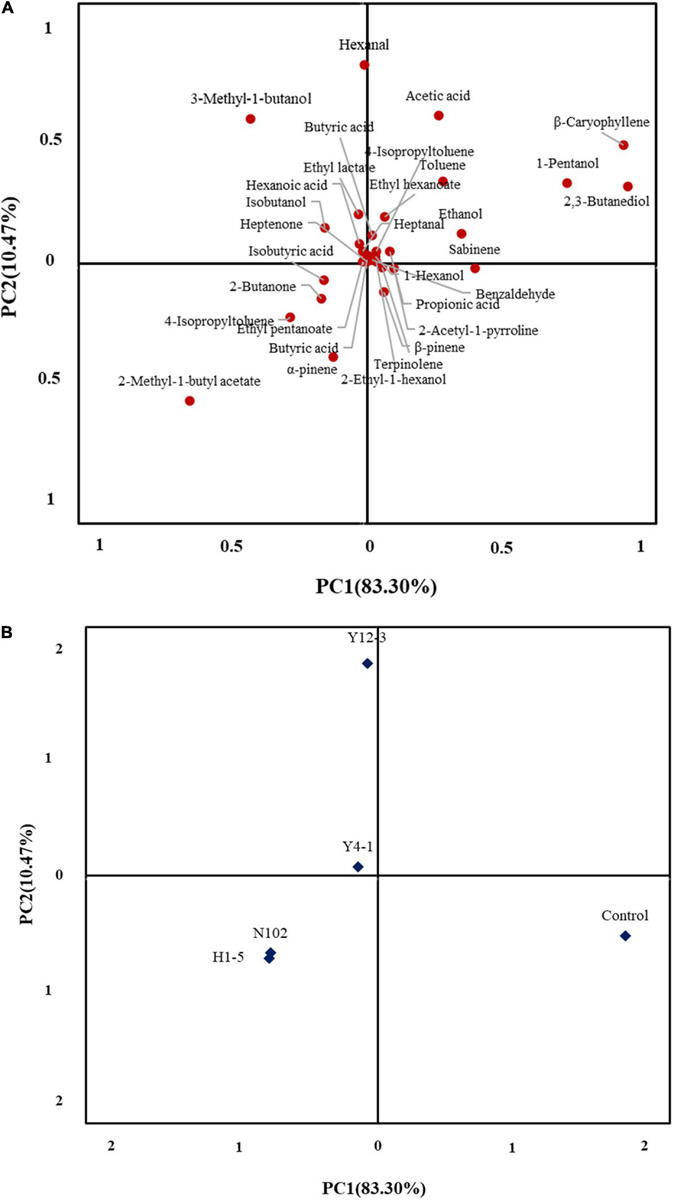
Principal component analysis loading plot **(A)** of volatile compound and principal component analysis score plot **(B)** of dry fermented sausages inoculated with different starter cultures.

In an attempt to further understand the difference in the volatile profile between the different inoculated strains of sausage, a total of 33 significantly different volatiles between two batches of sausages were used for the PCA. The first two principle components explained 83.30 and 10.47% of the overall variance, respectively (Figure 7). The first principal component (PC1) was the most important variable and positively correlated with most of the volatile compounds except for ten kinds of volatile compounds, 2-methyl-1-butyl acetate, 4-isopropyltoluened and 3-methyl-1-butanol. As shown in Figure 7B, inoculate lactobacillus, yeast, and control batches of sausages were well-differentiated along PC1.

### Sensory Evaluation

**FIGURE 8:**
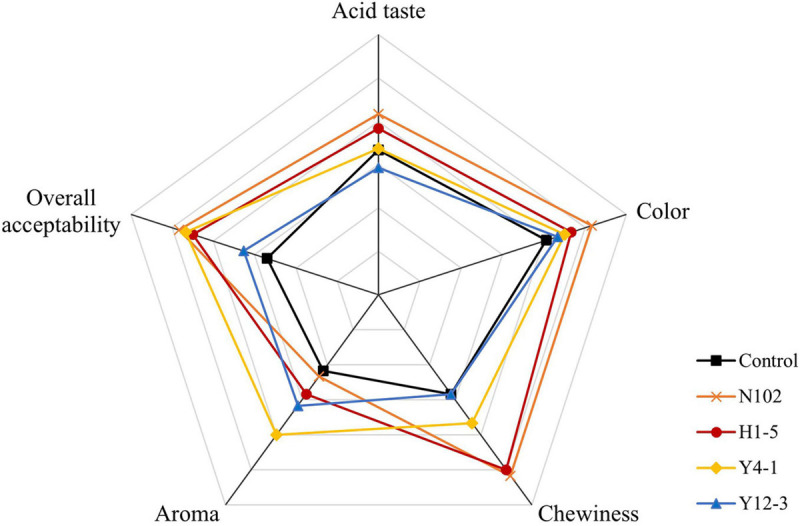
Sensory evaluation of the fermented sausages with different starter cultures.

The sensory scores, including color, aroma, chewiness, acid taste, and overall acceptability, are presented in Figure 8. The acidity of sausages in batches N102 and H1-5 is significantly higher than other batches. The sensory evaluation team members generally think that the Y4-1 batch of sausages is more aromatic (Fadda et al., 1999). The aroma scores of the Y4-1 batch of sausages were significantly higher than the other batches, which also correspond to the analysis results of volatile compound analysis. Compared with the control batch, the overall acceptability of sausages inoculated with starter was improved to varying degrees.

## Discussion

Fermented sausages are the result of biochemical, microbiological, physical and sensorial changes occurring in a meat mixture during ripening in defined conditions of temperature and relative humidity (Villani et al., 2007). Starter cultures are crucial in regulating the quality of fermented sausages. In this study, *L. delbrueckii* N102, *L. sakei* H1-5, *D. hansenii* Y4-1, and *W. anomalus* Y12-3 were selected from traditional fermented foods and inoculated into fermented sausages as starter cultures. The aim was to investigate the effects of different types of starter cultures on the microbial population, fat oxidation, lipolysis, proteolysis, and flavor compounds of the fermented sausage.

The decrease in pH of fermented sausages is due to the production of various organic acids such as lactic acid and acetic acid by LAB (Ammor and Mayo, 2007). Lower pH can inhibit the growth of harmful bacteria and contribute to product safety and extend the shelf life of the product (Osterlie and Lerfall, 2005; Flores and Toldra, 2011). The pH of the sausages inoculated with LAB was significantly lower than that of the control (*P* < 0.05), indicating that *L. delbrueckii* N102 and *L. sakei* H1-5 had strong acid-producing ability and produced more organic acids. This means that *L. delbrueckii* N102 and *L. sakei* H1-5 may help enhance the safety of dry fermented sausages. The pH rise in the middle and late stages of processing is mainly due to the action of microbes and enzymes of the meat tissue, which produces some basic ammonia and amines in the fermented sausage. However, yeast can raise the pH by using lactic acid (Flores et al., 2015). The LAB in each batches are the dominant bacteria, which is consistent with the results of Casaburi et al. (2016). It was found by microbial counts that the number of *Staphylococci* and *Micrococci* in the N102 and H1-5 batches were lower than that of the other batches, probably because *L. delbrueckii* N102 and *L. sakei* H1-5 reduced the pH and inhibited its growth (Casaburi et al., 2008). LAB ensure the safety of the product by reducing the pH of the sausage to inhibit the growth of *Enterobacteriaceae*.

Changes in the state of water in sausages affect product stability and shelf life and sensory qualities such as texture and juiciness by affecting microbial growth and enzyme activity (Fernández et al., 2000; Olivares et al., 2010). In meat products, bound water is an important part of the macromolecule compound; the immobilized water is located in the myofibril network, and the free water is outside the myofibril network (Bertram et al., 2004; Straadt et al., 2007). The change of the immobilized water and the free water is due to the effect of drying rather than fermentation; therefore, their content drops significantly during the late drying period. In sausage processing, the proportion of bound water increases because the structure of the protein changes due to oxidation, and muscle protein is hydrolyzed by bacteria or enzymes (Berardo et al., 2015).

*Lactobacillus delbrueckii* N102 and *L. sakei* H1-5 showed a strong ability to degrade sarcoplasmic proteins. This conclusion is consistent with the findings of Fadda et al. (1999), which showed that strains of *L. curvatus* and *L. sakei* were capable of hydrolyzing 97, 45, 37, and 26 kDa sarcoplasmic fractions. Protein degradation is one of the main reactions in the process of fermented sausages. It is generally believed that cathepsins play a major role in initiating proteolysis, while microbial enzymes play a weak role and play a role mainly in the late stage of sausage ripening (Berardo et al., 2017); of course, sausage processing also affects protein degradation. Due to the strong hydrolysis of protein by muscle and microbial peptidase, a large number of peptides and free amino acids are produced, which can be involved in the production of fermented sausage flavor. Many free amino acids are precursors to flavoring substances or are themselves flavoring substances. For example, glutamic acid contributes to umami, and the increase in content may be due to deamination of glutamine (Demasi et al., 1990); alanine contributes to sweetness (Ordonez et al., 1999). Branched-chain amino acids (valine, leucine, isoleucine) play an important role in the formation of flavor (Chen et al., 2016), and their content is significantly increased in all samples (*P* < 0.05), of which the leucine content in batch inoculated with H1-5 is the highest. The batch inoculated with *L. sakei* H1-5 has the highest levels of leucine and alanine which is consistent with the study of Flores and Toldra (2011). They believe that *L. sakei* has high exopeptidase activity, and it can produce a large amount of free amino acids (mainly leucine and alanine). The arginine content is reduced compared to day 0 because it can be utilized by the arginine deiminase (ADI) pathway (Mainar et al., 2017; Zagorec and Champomier-Verges, 2017). Taurine has excellent antioxidant activity (Gallego et al., 2018), which is highest in the H1-5 batch. In addition, hydrophobic amino acids (Ala, Phe, Val, Pro, Gly, Leu, and Ile), Glu, and His may also have antioxidant activity (Gallego et al., 2018). At the end of ripening, the dipeptides including carnosine and anserine were produced and have significant biological activity, which are higher than 200 mg/100 g, and the carnosine content of batch inoculated with N102 is significantly higher than that of the control (*P* < 0.05).

Endogenous enzymes found in meat tissues, such as lipases, esterases, and phospholipases, play an important role in the lipolysis process. Acid lipase activity can be activated by reducing water activity and increasing salt content (Motilva and Toldra, 1993). At lower pH, endogenous enzymes in muscle tissue are more efficient at degrading fat (Zanardi et al., 2004). The content of free fatty acids is affected by many factors, such as raw meat, processing time, and ingredients, so sausages release different free fatty acids due to various differences (Lorenzo and Franco, 2012). In addition, the FFA composition ratio of the fat tissue of all sausages was similar at the end of fermentation. In other words, during the fermentation process, bacterial fermentation increased the degree of hydrolysis of fats and oils and produced FFAs, but did not change the hydrolysis mode (Chen et al., 2016). It has been reported that hydrogen peroxide is produced by LAB metabolism, which also caused the lipid oxidant to increase. In our experiment, the TBARS value of the batch inoculated with Y4-1 and Y12-3 was lower than that of the other batch, indicating that the yeast may have an inhibitory effect on lipid oxidation. Meanwhile, Flores et al. (2004) found that the fermentation of sausage by inoculation with *D. hansenii* inhibited the oxidation of lipids and facilitated the formation of ethyl esters.

Flavor is one of the important qualities of fermented sausage. The carbohydrates in fermented sausages are metabolized by LAB to produce organic acids such as lactic acid, acetic acid, formic acid, propionic acid, butyric acid, and 3-methyl-butyric acid. Yeast in fermented sausages promotes the drying process of sausages (Lucke, 2000), and it also has proteolytic and lipolytic activities that affect the flavor of the product (Flores et al., 2004). The 2,3-butanediol was detected in all five batches of sausages with high content. Most alcohols are derived from the metabolism of carbohydrates by microorganisms such as ethanol and 2,3-butanediol, which are less stable, but can form diacetyl with 3-hydroxy-2-butanone to improve the overall flavor of the sausage (Mateo et al., 1996). Martin et al. (2003) reported that high concentrations of cyclic alcohol and aromatic alcohol can be produced when inoculating yeast. This result is consistent with the effect of strains Y4-1 and Y12-3 on the production of alcohols. Among the acid substances in all batches, the content of acetic acid is the highest, which is mainly formed by the metabolism of carbohydrates by LAB. In addition, the metabolism of fat and amino acids also produces acetic acid. Hexanoic acid content of the batch Y4-1 was the highest, probably due to the higher ethanol content of the Y4-1 batch reacting with butyric acid to form hexanoic acid. The batch inoculated with H1-5 detected higher isovaleric acid, which may be derived from the degradation of amino acids, which is consistent with H1-5 strains contributing to the release of amino acids. Acidic substances are important and representative flavor substances in fermented sausages and play an important role in the formation of esters. At the same time, the acid substance can improve the flavor complexity of the product and promote the fermentation sausage to form its unique sensory flavor characteristics. The source of aldehydes is mainly the oxidation of fats. The detection amount of aldehydes in the y4-1 inoculation batch was lower, especially hexanal and nonanal, which was corresponding to the results of TBARS discussed above, further confirming that Y4-1 may have antioxidant activity. Some branched aldehydes are derived from the Strecker degradation reaction of the corresponding amino acids and microbial action and the aldehydes of C5-C9 usually come from fat oxidation and have a fatty odor. Ketones contribute to lactic aroma notes, mainly due to the butter aroma produced by 2,3-butanedione, as well as other aroma notes such as mushroom and herbs. The 3-hydroxy-2-butanone and 2,3-octanedione detected in the HG sample were linked to citrate and lactose metabolism through the action of LAB and could also be generated through amino acid catabolism (Kieronczyk et al., 2004; Hu et al., 2020). The ester substance is necessary for the formation of the flavor of the fermented sausage, as the ethyl ester substance imparts a fruity and creamy aroma to the product, which is an important substance for promoting the formation of the flavor of the fermented sausage. Ethyl esters, usually present in fermented meat products, may arise from the action of inoculated yeast strains. The first reported effect on VOCs and aroma by yeast inoculation (*D. hansenii*) in fermented sausages indicated the promotion of ethyl ester compounds. Jesus Andrade et al. (2010) inoculated different *D. hansenii* strains isolated from dry cured hams in dry fermented sausages (Jesus Andrade et al., 2009) to study the effect on VOCs production. Similarly, Cano-Garcia et al. (2014b) believe that *D. hansenii* strains contribute to the esters in sausages. As the result of volatile compound analysis and principal component analysis showed, the flavor characteristics of LAB and yeast are obviously different. The strains Y4-1 and Y12-3 may contribute to the formation of esters, Yeast y4-1 and y12-3 can promote the esters formation such as ethyl lactate, ethyl hexanoate. Due to the presence of some pyruvate decarboxylases and alcohol dehydrogenases in yeasts (Dura et al., 2004), the alcohol content of the batches inoculated with yeast is higher. This aliphatic alcohol has not been described as essential in the aroma development of dry-cured meat products but it is the precursor of several esters (Molimard and Spinnler, 1996). This may be the reason why yeast promotes the production of esters. LAB were more inclined to produce butyric acid and isobutyric acid, which are produced through carbohydrate metabolism. The sensory analysis results also show that *D. hansenii* Y4-1 can enhance the flavor quality of dry fermented sausages. Compared with LAB, yeast, especially Y4-1, shows that the content of aldehyde compounds produced is lower, and the overall volatile flavor components are more abundant. This indicates that y4-1 may have the effect of inhibiting lipid oxidation and promoting the formation of dry fermented sausage flavor.

In conclusion, based on the understanding of the effect of single starter on the quality of dry fermented sausage, the functional differences between strains in sausage were further clarified, and the two starter cultures of LAB and yeast have their own advantages in acid production, bacteriostasis, protein degradation, antioxidant and flavor substance formation, which cannot be completely replaced by each other. However, considering the influence of strain on free amino acids and flavor, *L. sakei* H1-5 is better than *L. delbrueckii* N102. In addition, considering the long-term storage time of the product, antioxidant is also an important quality factor. Therefore, *D. hansenii* y4-1 is better than *W. anomalus* y12-3, which can be used for subsequent dry fermented sausage production and further sales. Of course, considering the combination of factors, it is necessary to further explore the overall impact of LAB and yeast cooperatively on the quality of salami sausage.

## Data Availability Statement

The original contributions presented in the study are included in the article/supplementary material, further inquiries can be directed to the corresponding author/s.

## Author Contributions

YL designed and drafted the manuscript. ZW carried out the physicochemical properties test and sausage making. KY performed the analysis of microbial population’s part. QY performed the analysis of volatile flavor compounds. ZY carried out the TBARS test. HL and JL provided helpful feedback and revised the manuscript. JW assisted in securing funding and managed the project. All authors contributed to the article and approved the submitted version.

## Conflict of Interest

The authors declare that the research was conducted in the absence of any commercial or financial relationships that could be construed as a potential conflict of interest.
